# The Influence of Pharmacological Preconditioning with Sevoflurane on Incidence of Early Allograft Dysfunction in Liver Transplant Recipients

**DOI:** 10.1155/2012/930487

**Published:** 2012-08-09

**Authors:** Andrei F. Minou, Alexander M. Dzyadzko, Aliaksei E. Shcherba, Oleg O. Rummo

**Affiliations:** ^1^Department of Anesthesiology and Critical Care, Republican Center of Organ and Tissue Transplantation, Semashko Street 8, 220116 Minsk, Belarus; ^2^Department of Transplantology, Republican Center of Organ and Tissue Transplantation, Semashko Street 8, 220116 Minsk, Belarus

## Abstract

*Background*. Pharmacological preconditioning is one of the tools used to diminish preservation injury. We investigated the influence of sevoflurane preconditioning of liver grafts on postoperative graft function. *Methods*. Consecutive 60 deceased brain donors were randomized into sevoflurane group or control group. In sevoflurane group donors were treated with endexpiratory 2,0 volume% of sevoflurane during procurement. Primary endpoint was postoperative liver injury. Secondary endpoint was incidence of early allograft dysfunction (EAD). *Results*. The groups were not different in median DRI, donor age, graft steatosis, and MELD score. Peak AST and ALT levels were lower in sevoflurane group than in control group: 792 and 1861 (*P* = 0, 038) for AST and 606 and 1191 for ALT (*P* = 0, 117). Incidence of EAD was 16,7% in sevoflurane group and 50% in control group (Fisher test, *P* = 0, 013). In subgroups without steatosis preconditioning with sevoflurane did not have influence on incidence of EAD. In subgroups with mild and moderate steatosis incidence of EAD was lower in recipients of liver grafts treated with sevoflurane. *Conclusions*. Preconditioning with sevoflurane during organ procurement improves graft function by lowering incidence of early allograft dysfunction, particularly in recipients of steatotic liver grafts.

## 1. Introduction

In order to accommodate the growing list of patients awaiting liver transplantation, the transplant community has increased efforts to expand the donor pool by utilization of extended criteria donor organs [1, 2], which include organs distinguished by hepatic steatosis, old donor age, prolonged cold ischemia, or donation after cardiac death. These grafts are susceptible to preservation injury and as a consequence are prone to a higher incidence of early allograft dysfunction (EAD) [3]. Moreover, severe ischemia/reperfusion injury (IRI) significantly impacts transplantation outcome because it is a major risk factor for both early graft failure and late chronic allograft dysfunction.

Pharmacological preconditioning is one of the tools used to diminish preservation injury. Although volatile anesthetics and propofol have been studied to attenuate injury in liver resections with inflow occlusion [4, 5], pharmacological preconditioning with sevoflurane of human liver grafts from deceased brain donors has not yet been described. The purpose of this study was to evaluate the influence of sevoflurane preconditioning of liver grafts from deceased brain donors on postoperative graft function in patients undergoing liver transplantation.

## 2. Materials and Methods

Deceased brain liver donors were assessed for study eligibility to enroll overall number of 60 donors (30 in each group). Exclusion criteria were age less than 18 years and utilization of the procured graft for retransplantation or reduced size liver transplantation. Between November 2010 and December 2011 consecutive 67 deceased brain donors were assessed of whom 7 were excluded as they fell under exclusion criteria. Enrolled donors were randomized at the beginning of the procurement procedure into a sevoflurane group (inhaled anesthesia with sevoflurane) or a control group (without any volatile anesthetic). All other medication and surgical management was the same. The randomization sequence without any stratification was generated by computer and sealed with consecutively numbered envelopes providing concealment of random allocation. The study was approved by local ethics committee. Written informed consent was obtained from all recipients before liver transplantation.

At the beginning of procurement procedure all donors received bolus of 3 *μ*g/kg fentanyl to blunt spinal reflexes and bolus of 12 mg pipecuronium bromide for muscle relaxation. Electrocardiogram, arterial oxygen saturation, central venous pressure, and radial arterial blood pressure were monitored routinely. To maintain mean arterial pressure higher than 60 mm Hg infusion of norepinephrine 0,05–0,15 *μ*g/kg/min was administered as indicated. In sevoflurane group pharmacological preconditioning with end-expiratory sevoflurane of 2,0 volume% in mixture of oxygen and air (FiO_2_ = 0,4) was performed for the entire procedure of organ procurement. In control group donors inhaled only mixture oxygen and air (FiO_2_ = 0,4) without any volatile anesthetic.

All organ procurement procedures were performed in standardized manner by 3 experienced transplant surgeons, who were blinded to randomization. After laparotomy in all donors liver specimen was taken for evaluation for the presence of steatosis. Liver biopsies were evaluated by a single pathologist for the presence of macrovesicular steatosis. Using haematoxylin and eosin-stained sections, the degree of macrovesicular steatosis was graded as absent (0%), mild (1–30%), or moderate (31–60%) based on the percentage of hepatocytes with fat droplets.

All donor organ allografts were implanted by caval replacement technique with bicaval anastomoses. The anesthetic technique was the same in all recipients. Maintenance of anesthesia was obtained with sevoflurane (the minimal alveolar concentration was between 0,7 and 0,9). Fentanyl was given as continuous infusion. During a hepatic phase and after reperfusion norepinephrine 0,1–0,3 *μ*g/kg/min was used in all recipients to maintain mean arterial pressure at more than 60 mm Hg. Patients were ventilated with a fraction of inspired oxygen of 0,4 to 0,6, tidal volumes of 7 to 10 mL/kg, and a positive end-expiratory pressure of 0 to 5 cm H_2_O. Transfusion triggers were similar, with a target hematocrit of 25 to 30. Fresh frozen plasma, cryoprecipitate, and platelet concentrate were administered at the discretion of the attending anesthesiologist and according to results of thromboelastometry. All patients received intraoperative 500 mg methylprednisolone as induction of immunosuppression. Transplant surgeons and anesthesiologists were blinded to randomization.

Primary endpoint was postoperative liver injury assessed by peak serum values of alanine aminotransferase (ALT) and aspartate aminotransferase (AST). Secondary endpoint was incidence of early allograft dysfunction (EAD) defined as the presence of one or more of the following criteria: bilirubin greater or equal 10 mg/dL on day 7, international normalized ratio (INR) greater than or equal to 1,6 on day 7, and ALT or AST greater than 2000 IU/L within the first 7 days after liver transplantation [6]. We performed limited number of subgroup analyses to assess the influence of degree of macrovesicular steatosis on protective effect of pharmacological preconditioning with sevoflurane.

Group sample size was calculated based on two assumptions. In our previous experience the mean postoperative peak levels of AST and ALT were 2532 ± 1013 IU/L and 1561 ± 625 IU/L, respectively. We expected that pharmacological preconditioning with sevoflurane could produce 30% reduction in peak levels of liver transaminases, as it was observed by Beck-Schimmer et al. [4]. Thus, to achieve a 30% reduction in peak levels of transaminases with an *α* error of 0,05 and a power 0,80, 29 patients were needed in both groups. The Shapiro-Wilk test was used to check for normal distribution. Nonparametric data were expressed as median (interquartile range) and parametric data as mean ± SD. Group means were compared using Mann-Whitney *U*-test or Student's *t*-test as appropriate. Categorical variables were compared using two-tailed Fisher's exact test. Significance was defined as *P* < 0,05. Calculations were made using SPSS 18.0 software (SPSS Inc., Chicago, IL, USA).

## 3. Results

Thirty liver grafts from deceased brain donors were included in each group. Donor demographics, as well as cold and warm ischemia time, percentage of steatosis, and donor risk injury (DRI) score (calculated as described by Feng et al. [7]) are presented in Table 1. None of these parameters were statistically different.

Table 2 shows the recipient characteristics: age, MELD score, baseline levels of ALT, and AST. There were no significant differences in recipient characteristics between sevoflurane and control groups. No one in both groups had severe comorbidities such as coronary artery disease, chronic obstructive pulmonary disease, or diabetes mellitus.

The degree of IRI of the liver was assessed by postoperative peak serum ALT and AST levels. The peak of the transaminases occurred between 24 and 48 hours after surgery. The peak levels of AST and ALT were lower in sevoflurane group compared to the control group, but the difference was statistically significant only for peak level of AST (Table 3). The incidence of early allograft dysfunction (EAD) was lower in sevoflurane group (16,7%) compared with control group (50%). No patient experienced primary graft nonfunction in both groups. Despite higher incidence of EAD in control group, there were no significant differences in length of ICU and hospital stay between two groups (Table 3).

 We performed limited number of subgroup analyses to further investigate the influence of degree of macrovesicular steatosis on the protective effects of pharmacological preconditioning with sevoflurane. The two groups were almost identical with respect to numbers of liver grafts without steatosis, with mild and moderate macrovesicular steatosis (Table 4).

 The pharmacological preconditioning with sevoflurane did not have influence on the IRI of grafts without macrovesicular steatosis. There were no significant differences in peak levels of aminotransferases between the two subgroups. In subgroups with mild macrovesicular steatosis the peak values of liver transaminases were lower in recipients of grafts treated with sevoflurane compared to recipients of grafts without any preconditioning, but the differences were not statistically significant. In subgroups with moderate macrovesicular steatosis the peak levels of AST and ALT were lower in recipients of grafts with sevoflurane preconditioning compared to recipients of grafts without preconditioning, but the difference was statistically significant only for peak level of AST (Table 5).

Effect of sevoflurane preconditioning on graft function depended on the degree of macrovesicular steatosis. In subgroups without macrovesicular steatosis pharmacological preconditioning with sevoflurane did not have influence on incidence of EAD. In subgroups with mild and moderate macrovesicular steatosis incidence of EAD was lower in recipients of liver grafts treated with sevoflurane, but the difference was statistically significant only in subgroup with mild steatosis (Table 6).

## 4. Discussion

We evaluated the influence of sevoflurane preconditioning of liver grafts from deceased brain donors on postoperative graft function in patients undergoing liver transplantation. This randomized controlled trial demonstrated the protective effects of pharmacological preconditioning with sevoflurane. Reperfusion injury assessed by peak serum levels of transaminases was attenuated in sevoflurane group. Preconditioning with sevoflurane improved graft function by lowering the incidence of early allograft dysfunction. The observed protective effects were seen only in recipients of grafts with macrovesicular steatosis and were absent in recipients of grafts without macrovesicular steatosis. Our data are consistent with results of the study by Beck-Schimmer et al. [4], in which protective effects of preconditioning with sevoflurane on IRI were more pronounced in patients with liver steatosis.

Postoperative liver graft function depends on many factors related to donor and recipients characteristics. The DRI developed by Feng et al. [7] is a continuous scoring system, which includes only donor and transplant parameters found to significantly influence outcomes after liver transplantation. The DRI was also validated for use within Eurotransplant region [8]. In our study the two groups were comparable with respect to DRI score and some parameters included in DRI: donor age and cold ischemia time. There were no differences between two groups in characteristics not included in DRI, but which could have influence on graft function: warm ischemia time and percentage of macrovesicular steatosis. In 2010 Olthoff et al. [6] validated a current definition of EAD. In their study in the multivariable analysis only the age of the donor and MELD score were significantly associated with EAD. Neither donor age nor MELD score was statistically different in our study.

The development of strategies to counteract IRI of the liver is a major challenge in liver surgery and transplantation. Some of clinical strategies reported to ameliorate IRI in liver transplantation include donor treatment with steroids [9], caspase inhibition [10], ATG induction therapy [11], donor organ flush with calcineurin inhibitor [12], nitric oxide (NO) inhalation [13], and infusion of the donor liver with rPSGL-Ig [14]. Pharmacological preconditioning of liver grafts with sevoflurane is a new strategy, which can easily be applied during organ procurement procedure and lacks of serious adverse effects.

Despite the fact that preconditioning with sevoflurane is a new strategy for liver transplantation, it has been examined in other clinical situations, including liver surgery. There is growing evidence that pharmacological preconditioning with volatile anesthetics may provide a new and easily applicable therapeutic option to protect the liver from IRI. In the study by Imai et al. [15] isoflurane, sevoflurane, and halothane reduced IRI in isolated perfused rat liver when administered during the reperfusion phase; however, they did not reduce injury when administered only during ischemia. Imai et al. [15] suggested that volatile anesthetics might protect the fasted liver from early, neutrophil-independent IRI by acting during the reperfusion phase. The results of study by Ishida et al. [16] showed that the extent of the hepatic IRI seen under sevoflurane anesthesia in pigs did not differ significantly from that seen under isoflurane, as judged from measurements of a number liver damage markers over a 240 min reperfusion period. The study conducted by Bedirli et al. [17] suggested that clinically relevant concentrations of sevoflurane given before, during and after hepatic ischemia protected the liver in rats against IRI, whereas the effects of isoflurane on hepatic IRI were not notable. The study by Beck-Schimmer et al. [4] showed that sevoflurane preconditioning was protective against IRI during liver resection. Sevoflurane preconditioning was shown to prevent hepatic injury, defined by transaminase levels, and improve clinical outcome. In the volatile preconditioning group, the expression of inducible nitric oxide synthase upon reperfusion significantly increased compared with the baseline value, which points to a possible protective role of nitric oxide in pharmacological preconditioning. The observed protective effects were more pronounced in patients with liver steatosis. However, Song et al. [5] compared liver function after hepatectomy with inflow occlusion between sevoflurane and propofol anesthesia and found no significant differences in postoperative liver function, as measured by serial transaminase levels, or in clinical outcomes. Patients with biopsy-proven cirrhosis did not have worse postoperative liver function than those without cirrhosis likely because the period of ischemia was too short to have significant impact on liver function. Perhaps, longer ischemic stress could reveal differences between sevoflurane and propofol anesthesia. The study by Ko et al. [18] suggested better postoperative hepatic and renal function tests with desflurane than sevoflurane at equivalent dose of 1 minimum alveolar concentration in living donors undergoing right hepatectomy.

Our study had several limitations. Although the study suggested statistically significant differences between sevoflurane and control groups with respect to incidence of EAD, our study was underpowered as sample size, especially in subgroup analysis, and was relatively small. Therefore interpretation of subgroup analysis needs to be done carefully as reproducibility of these findings is low. Despite the fact that the statistical significance of our data is limited by the sample size, we believe that this is the first clinical trial to demonstrate a protective effect of sevoflurane preconditioning during organ procurement on graft function in liver transplantation.

## 5. Conclusions

Our data indicate that there is a beneficial effect of preconditioning with sevoflurane on liver graft function. Pharmacological preconditioning with sevoflurane during organ procurement improves graft function by lowering incidence of early allograft dysfunction, particularly in recipients of steatotic liver grafts. 

## Figures and Tables

**Table 1:** Donor characteristics.

	Sevoflurane group	Control group	Mann-Whitney, *P* value
Donor age, y	32 (24–46)	39 (25–46)	0,515
Weight, kg	75 (63,5–80)	75,5 (70–85)	0,596
BMI, kg/m^2^	24,2 (22,8–25,9)	24,8 (21,9–26,8)	0,744
Cold ischemia time, min	455 (365–565)	465 (400–585)	0,329
Warm ischemia time, min	62,5 (60–70)	67,5 (60–70)	0,623
Percentage of macrovesicular steatosis, %	15 (0–20)	10 (0–20)	0,922
DRI score	1,19 (1,03–1,25)	1,23 (1,11–1,38)	0,186

Data are as median (interquartile range). BMI: body mass index, DRI: donor risk injury.

**Table 2:** Recipient characteristics.

	Sevoflurane group	Control group	Mann-Whitney, *P* value
Recipient age, y	44 (28–55)	49 (34–55)	0,865*
MELD score	20 (15–27)	17 (14–26)	0,340*
Baseline AST, IU/L	86 (32–154)	90 (34–171)	0,955*
Baseline ALT, IU/L	62 (17–126)	63 (20–220)	0,985*
Beta-blocker therapy, y/n	17/13	15/15	0,796**

Data are as median (interquartile range).

*Mann-Whitney test, **Fisher's exact test, two tailed.

**Table 3:** The influence of pharmacological preconditioning with sevoflurane on graft function and length of ICU and hospital stay.

	Sevoflurane group	Control group	*P* value
Peak AST, IU/L	792 (481–1436)	1861 (519–3590)	0,038*
Peak ALT, IU/L	606 (344–892)	1191 (392–2137)	0,117*
Incidence of EAD, %	16,7 (5 of 30)	50,0 (15 of 30)	0,013**
Length of ICU stay, d	6 (5–8)	6 (4–9)	0,655*
Length of hospital stay, d	18 (14–22)	18 (15–26)	0,833*

Data are as median (interquartile range). AST: aspartate aminotransferase, ALT: alanine aminotransferase, EAD: early allograft dysfunction, ICU: intensive care unit.

*Mann-Whitney test, **Fisher's exact test, two tailed.

**Table 4:** Number of grafts without and with mild and moderate macrovesicular steatosis.

	Macrovesicular steatosis		
	None	Mild	Moderate
	(0%)	(1–30%)	(31–60%)
Sevoflurane group, *n*	9	16	5
Control group, *n*	9	15	6

**Table 5:** The influence of degree of macrovesicular steatosis on IRI of grafts with and without pharmacological preconditioning.

Macrovesicular steatosis	AST		ALT	
	Sevoflurane	Control	Sevoflurane	Control
None (0%)	669 (276–1327)	759 (315–1021)	421 (285–974)	413 (222–603)
Mild (1–30%)	825 (515–1654)	2571 (524–3493)	576 (363–861)	1666 (481–2642)
Moderate (31–60%)	979 (658–2267)*	4002 (2322–8601)	757 (428–1776)	1711 (940–3474)

Data are as median (interquartile range). AST: aspartate aminotransferase, ALT: alanine aminotransferase.

*P < 0,05 (Mann-Whitney *U*-test) versus control group.

**Table 6:** The influence of degree of macrovesicular steatosis on protective effects of sevoflurane preconditioning.

Macrovesicular steatosis	Incidence of EAD, %		Fisher's exact test, two-tailed *P* value
	Sevoflurane group	Control group	
None (0%)	11,1 (1 of 9)	11,1 (1 of 9)	1,000
Mild (1–30%)	18,8 (3 of 16)	60,0 (9 of 15)	0,029
Moderate (31–60%)	20,0 (1 of 5)	83,3 (5 of 6)	0,080

EAD: early allograft dysfunction.
